# Morbidity and Mortality Trends in Preterm Neonates at the Limits of Viability: Retrospective Observations from One Greek Hospital

**DOI:** 10.3390/life15050708

**Published:** 2025-04-27

**Authors:** Dimitra Maria Apostolidi, Maria Kapetanidi, Eleni Vretou, Antigoni Sarantaki, Katerina Lykeridou, Grigorios Karampas, Athina Diamanti, Maria Vlachou, Nikoleta Pantelaki, Anna Deltsidou, Christina Nanou, Stavroula Charoni, Panagiota Katti, Aikaterini Fotiou, Iraklis Salvanos, Dimitra Metallinou

**Affiliations:** 1 Department of Midwifery, School of Health and Care Sciences, University of West Attica, 122 43 Athens, Greece; di.mi98@hotmail.com (D.M.A.); midw20681034@uniwa.gr (M.K.); midw20681014@uniwa.gr (E.V.); esarantaki@uniwa.gr (A.S.); klykeridou@yahoo.gr (K.L.); adiamanti@uniwa.gr (A.D.); mvlachou@uniwa.gr (M.V.); npantelaki@uniwa.gr (N.P.); adeltsidou@uniwa.gr (A.D.); nanouxv@uniwa.gr (C.N.); 2 Second Department of Obstetrics and Gynaecology, Aretaieio Hospital, National and Kapodistrian University of Athens, 115 28 Athens, Greece; karampasgrig@gmail.gr; 3 Neonatal Intensive Care Unit, Helena Venizelou General & Maternity Hospital, 115 21 Athens, Greece; stavroulacharoni@hotmail.com (S.C.); pan.katti@yahoo.com (P.K.); katerinifg@hotmail.gr (A.F.); iraklis.salvanos@yahoo.gr (I.S.)

**Keywords:** morbidity, mortality, preterm neonates, infants, limits of viability, neonatal intensive care unit, risk factors, survival predictors, observational study

## Abstract

The survival and health outcomes of extremely preterm neonates (PNs) remain a critical challenge in neonatal intensive care. This 5-year retrospective, observational study evaluated morbidity and mortality trends in PNs born at the limits of viability and identified survival patterns and associated risk factors. It was conducted from 2017 to 2022 on a dataset of PNs born between 22 + 0 and 26 + 0 weeks of gestation in a tertiary public hospital in Greece. A total of 73 PNs were included. The mortality rate was 56.2%. The median gestational age was 24.3 weeks, and the mean birth weight was 603.6 g. Survival improved significantly with a higher gestational age and birth weight. Respiratory distress syndrome was the most prevalent morbidity (71–94%), followed by late-onset sepsis (35.3%) and patent ductus arteriosus (29.4%). The use of antenatal corticosteroids and enteral feeding were associated with improved survival rates. Survivors required prolonged respiratory support and demonstrated better outcomes with early and adequate nutritional support. We conclude that the gestational age, birth weight, and effective respiratory and nutritional interventions are critical determinants of survival in neonates at the limits of viability. Enhancing neonatal care protocols with targeted interventions, such as antenatal corticosteroid use and evidence-based nutritional practices, could significantly improve outcomes in this vulnerable population.

## 1. Introduction

Preterm birth, defined as delivery before 37 completed weeks of gestation, is a major global health concern, contributing significantly to neonatal morbidity and mortality. In 2020, an estimated 13.4 million neonates were born preterm, making preterm birth complications the leading cause of death among children under five years of age, accounting for approximately 900,000 deaths in 2019 alone [1,2]. Despite significant advances in neonatal care, the survival and long-term outcomes of preterm neonates, particularly those born at the limits of viability, remain variable and challenging to predict.

Periviable neonates are at the greatest risk for mortality and long-term morbidities. Survivors of extreme prematurity frequently face a myriad of health complications, including respiratory distress syndrome (RDS), bronchopulmonary dysplasia (BPD), intraventricular hemorrhage (IVH), necrotizing enterocolitis (NEC), and long-term neurodevelopmental disabilities, such as cerebral palsy, visual impairments, and cognitive delays [3,4]. These outcomes not only affect the quality of life for the neonates and their families but also place a significant burden on healthcare systems globally [5,6].

Survival outcomes of periviable neonates vary widely depending on the geographic region, healthcare resources, and clinical practices. A recent meta-analysis showed that the survival rates increase with each additional week, from 7% at 22 weeks to 68% at 25 weeks among live births. However, outcomes are heavily influenced by resource availability. In high-income countries, neonatal interventions, such as mechanical ventilation, surfactant therapy, and antenatal corticosteroids, improve survival, with 31% of NICU-admitted neonates surviving at 22 weeks. In contrast, in low- and middle-income countries, survival at 22 weeks is only 10% due to the limited access to intensive care technologies, trained neonatal staff, and standardized treatment protocols. Survival rates have improved over time, reflecting upgrades in neonatal care, yet disparities among countries persist [7].

Although significant improvements in neonatal care have resulted in better survival rates for periviable neonates in high-resource settings, the impact of long-term morbidity remains substantial. These outcomes highlight the importance of early interventions and improved clinical protocols tailored to preterm neonates, particularly those born at the limits of viability [8,9]. However, despite the availability of global data, single-center studies provide invaluable insights into the specific factors influencing morbidity and mortality in local populations. Institutional practices, patient demographics, and access to healthcare resources can vary widely, affecting outcomes in ways that large-scale studies may not capture [10,11].

In Greece, neonatal resuscitation and intensive care for extremely preterm neonates often begin as early as 22 weeks of gestation, reflecting local clinical practices despite international guidelines generally discouraging active management before 23 weeks. These practices are influenced by the lack of established neonatal palliative care protocols within the Greek healthcare system, as documented in recent studies [12]. Unlike other countries where structured palliative care pathways exist for neonates born at the limits of viability, Greek neonatal units primarily follow an active therapeutic approach, with management decisions guided by institutional protocols, the overall neonatal clinical condition, and parental consultations. This approach underscores the urgent need for standardized neonatal palliative care guidelines in Greece to provide comprehensive support for neonates and families facing extremely preterm births.

This retrospective study aims to assess the morbidity and mortality of preterm neonates born at the limits of viability at a single tertiary hospital. By exploring the clinical outcomes of these neonates we seek to identify survival patterns, associated morbidities, and risk factors linked to adverse outcomes. Understanding these factors is crucial for guiding future improvements in neonatal care, optimizing the use of available resources, and informing clinical decision-making for this vulnerable population.

## 2. Materials and Methods

### 2.1. Study Design and Participants

This study is a 5-year retrospective, single-center, observational study conducted at a Neonatal Intensive Care Unit of a tertiary public hospital in Attica, Greece. It focused on the years 2017–2022.

This study included preterm neonates at the limits of viability. Due to the fact that a universal definition of the “limits of viability” is likely unattainable because of its variability across settings and communities, no clear definition has been established to date. Despite ongoing research on critically ill neonates, a precise scientific definition of the limits of viability has yet to be produced [13]. Given that Greece is a developed European country that enjoys certain economic advantages and access to advanced infrastructure, in this study we focused on extremely preterm neonates born between 22 and 26 weeks of gestation. The gestational age was determined based on obstetric records, including the last menstrual period or first-trimester ultrasound. We included all preterm neonates born in the hospital at that gestational age, including those who, despite resuscitation efforts, passed away in the delivery room or operating theater.

Patients or members of the public were not involved in the design, conduct, reporting, or dissemination plans of this research.

### 2.2. Data Collection

Data were extracted from the obstetric and neonatal records using a standardized data collection form, between August 2023 and February 2024. Information including maternal and neonatal characteristics and neonatal clinical outcomes was collected. Mortality was defined as death occurring before discharge from the NICU, as well as death in the delivery room or operating theater, as previously mentioned.

### 2.3. Ethical Considerations

The study protocol was reviewed and approved by the Institutional Review Board (IRB: 15285/07-07-2023), ensuring compliance with ethical standards for research involving human subjects. The requirement for informed consent was waived due to the retrospective design of this study, as it involved data already collected and did not directly impact patient care. To safeguard privacy, all patient data were anonymized, with identifying information removed to maintain strict confidentiality. This study adhered to the principles outlined in the Declaration of Helsinki, emphasizing respect for patient rights and the ethical use of medical data.

### 2.4. Statistical Analysis

Variables were first tested for normality using the Kolmogorov–Smirnov criterion. Normal distributed variables are expressed as mean ± standard deviation, while variables with a skewed distribution are expressed as median (interquartile range). Qualitative variables were expressed as absolute and relative frequencies. If the normality assumption was satisfied for the comparison of means between two groups, Student’s *t*-tests were used. The Mann–Whitney test was used for the comparison of continuous variables between two groups when the distribution was not normal. For the comparison of proportions, the chi-square test or Fisher’s exact test was used. A stepwise logistic regression analysis was used in order to find independent factors associated with neonatal mortality. Adjusted odds ratios (ORs) with 95% confidence intervals (95% CIs) were computed from the results of the logistic regression analyses. All reported *p* values are two-tailed. Statistical significance was set at *p* < 0.05 and analyses were conducted using SPSS statistical software (version 26.0).

## 3. Results

The study sample comprised 73 neonates born between 22 and 26 weeks of gestation, of which 41 died (56.2%). The mean maternal age was 31.1 years (SD = 7.1 years). Among the neonates, 54.8% were first-borns, 23.4% were second-borns, and 17.8% were third- or fourth-borns. The median gestational age was 24.3 weeks, with an interquartile range (IQR) of 22.9 to 25.2 weeks. The majority of pregnancies (82.2%) involved a single fetus and 53.4% of the neonates were male. Most neonates (52.1%) were born via vaginal delivery. Additionally, 8.2% of the mothers had undergone an in vitro fertilization treatment (IVF). Regarding prenatal corticosteroid administration, 37.4% of the neonates received complete therapy, while 24.7% received partial therapy. The median Apgar score was four at the first minute post-birth (IQR: 2–5), increasing to seven at the fifth minute (IQR: 4–8). For neonates whose head circumference was measured at birth, the mean was 22.1 cm (SD = 1.7 cm). The mean birth weight was 603.6 g (SD = 155.1 g). A significant majority (97.1%) of the neonates required resuscitation either in the operating or delivery room. Among these, 63.4% received NEOPUFF, 62% required intubation, and 16.9% received adrenaline. All information regarding maternal and neonatal characteristics is summarized in Table 1.

The clinical outcomes varied, with 23.3% achieving favorable outcomes, 20.5% requiring transfer to another hospital, and 56.2% experiencing mortality. Late-onset sepsis occurred in 11% of the neonates, while 42.9% experienced at least one episode of possible sepsis. Caffeine therapy was administered to 71.2% of the neonates, whereas inotropes were used in only 2.7% of the cases. Surfactant therapy was administered to the majority of neonates, with 30.1% receiving one dose, 27.4% receiving two doses, and 2.7% requiring three doses. However, 39.7% of the neonates did not receive surfactant therapy. Among neonates diagnosed with a patent ductus arteriosus (PDA), 71.4% were treated with paracetamol. Additionally, cases of NEC, specifically intestinal perforation, cerebral hemorrhage, periventricular leukomalacia (PVL), and retinopathy of prematurity (ROP), were observed. However, there were no cases of possible or confirmed NEC, grade III cerebral hemorrhage on the right side, grade II–IV PVL, or stage III ROP. Enteral nutrition was provided to 30.1% of the neonates, while 69.9% did not receive enteral feeding during hospitalization. Additional feeding data revealed that 41.7% of the neonates received mixed feeding, 39.9% were exclusively fed with breast milk (either maternal or donor), 16.7% received formula, and only one neonate (2.8%) received a special formula. At discharge, the surviving neonates had a mean weight of 2387.1 g (SD = 1495.4 g) and a mean head circumference of 31.8 cm (SD = 4.8 cm). Moreover, 15.4% of the neonates (N = 4) were diagnosed with hypotonia and 84.6% (N = 22) demonstrated a normal neurological status at the time of discharge. Table 2 displays the additional neonatal clinical characteristics during hospitalization.

**Figure 1:**
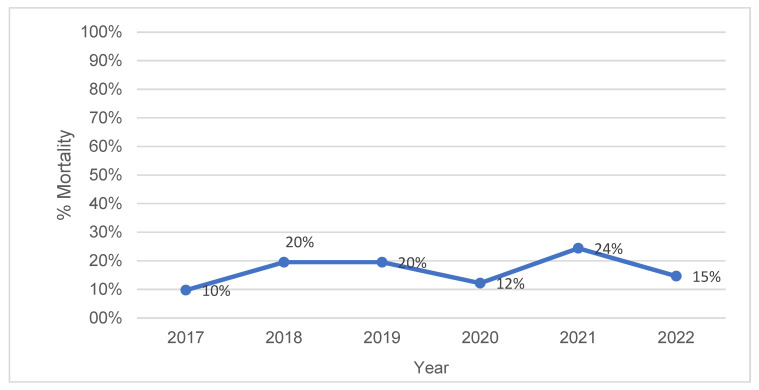
The mortality rates of preterm neonates over the 5-year study period (2017–2022).

**Figure 2:**
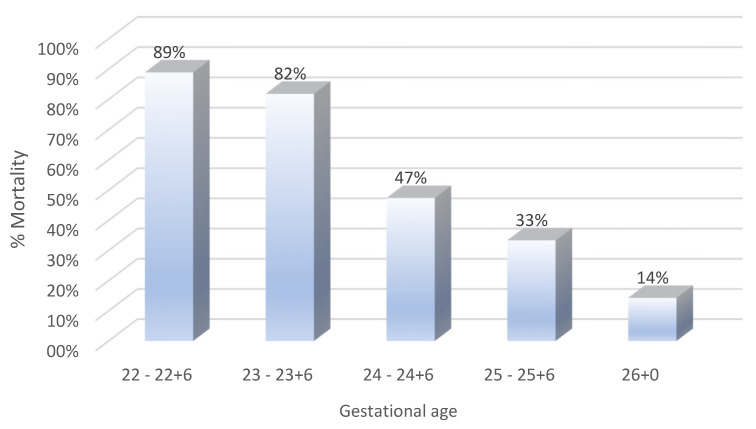
Mortality rates of preterm neonates stratified by gestational age.

The mortality rates of preterm neonates born at the limits of viability exhibited noticeable fluctuations over the 5-year period, ranging from a low of 10% to a high of 24% (Figure 1), while the stratification by the gestational age revealed a clear decline in mortality with an increasing gestational maturity (Figure 2).

The univariate analysis of the data, presented in Table 3, indicated that neonates who survived had a significantly greater gestational age compared to those who did not survive. Survivors also had higher birth weights and better health assessments, reflected in their five-minute Apgar scores. Moreover, surviving neonates required a longer duration of respiratory support, regardless of the method used, and a higher number of them also required red blood cell transfusions. The analysis also highlighted significant differences in outcomes based on the number of fetuses. In multiple gestations, the mortality rate was 30.8%, which was significantly lower than the 61.7% observed in singleton cases. The relationship between surfactant administration and neonatal outcomes was also significant. Mortality rates were markedly higher at 69% for neonates who received no surfactant and 63.5% for those given only one dose, compared to just 31.8% among neonates who received two or three doses. Additionally, 70.7% of neonates who did not receive enteral feeding did not survive, a significantly higher rate than the 22.7% mortality observed in those who were fed enterally.

The logistic regression analysis, conducted using the stepwise method, included independent variables related to both maternal and neonatal factors (Table 4). The model identified significant independent associations between the neonatal mortality, maternal age at delivery, and birth weight, indicating that a higher birth weight and greater gestational age significantly reduced the mortality risk. Furthermore, when the model focused solely on neonatal-related factors as independent variables, it identified the duration of conventional respiratory support and the gestational age as significant predictors of mortality (Table 5). Specifically, a longer duration of respiratory support and a higher gestational age were both associated with a reduced likelihood of neonatal mortality.

## 4. Discussion

This 5-year retrospective, single-center study provides valuable insights into the morbidity and mortality of preterm neonates born at the limits of viability (22–26 weeks of gestation), highlighting key factors influencing survival and health outcomes in this vulnerable population. Notably, to our knowledge, this is the first study of its kind conducted in Greece, offering a unique perspective on neonatal care in this region.

The mortality rates of preterm neonates at the limits of viability varied significantly, ranging from 10% to 24%, likely influenced by advancements in neonatal care, evolving clinical practices, and resource availability. Lower rates in some years (10–12%) may reflect improved protocols or favorable patient profiles, while higher rates (20–24%) could correspond to more complex cases or limited resources, emphasizing the need for continuous quality improvements in neonatal care and a deeper analysis of year-to-year differences to identify potential factors contributing to better survival outcomes. Furthermore, the median hospital stay in our study was 6 days (IQR: 1–58), reflecting substantial variability. The shorter stays (1 day) were largely due to early neonatal mortality, while the longer stays (up to 58 days) represented surviving neonates requiring prolonged intensive care. Additionally, 20.5% of neonates were transferred to other centers, further impacting the hospitalization duration. Although the institution is a tertiary care center with a fully equipped neonatal intensive care unit, these transfers were necessitated by the need for specific services not available on-site (e.g., retinal photocoagulation, neurosurgical interventions). In some instances, transfers also occurred due to the unavailability of a free incubator, despite the level of care offered, particularly during periods of high occupancy. All neonates were clinically stable at the time of transfer which allowed for the safe transport and appropriate continuation of care in specialized centers. These factors highlight the complex clinical course of extremely preterm neonates, where early mortality and interhospital transfers play a crucial role in shaping hospitalization patterns. Therefore, the reported hospital stay should be interpreted in the context of these influencing factors, rather than as an indicator of the standard neonatal care duration at this gestational age.

Mortality rates declined with an increasing gestational age (22–26 weeks), with 22-week neonates facing the highest mortality and 26-week neonates showing a significantly improved survival, highlighting the critical role of gestational maturity. However, the gestational age alone does not fully explain survival differences. In our study, the lower mortality in multiple gestations (30.8%) vs. singletons (61.7%) cannot be attributed to differences in gestational age, as our analysis revealed no statistically significant difference in the mean gestational age between the two groups (*p* = 0.826). This suggests that the survival advantage observed in multiple gestations was likely driven by perinatal management factors, such as closer prenatal monitoring, higher rates of antenatal corticosteroid administration, and planned cesarean deliveries, which may have helped reduce birth-related complications.

In addition, the findings of this study underscore the severe challenges of preterm neonates at the limits of viability, reflected in a 56.2% mortality rate, which is consistent with global trends influenced by gestational age, clinical practices, and resource availability. For instance, Zhu et al. [6] reported a 95.2% mortality at 22 weeks, with survival improving as the gestational age increases—a trend also seen in Younge et al. [5] and large European studies including EPICURE (UK) [14], MOSAIC (Europe) [15], and EXPRESS (Sweden) [16], which demonstrated that neonatal survival improves significantly beyond 24 weeks, reinforcing the critical role of gestational maturity in determining outcomes. Although our results align with existing survival patterns, the mortality rates observed in our study appear lower than those reported in some large national or multicenter studies, such as that of Edwards et al. [17]. This discrepancy is primarily due to our study being a single-center analysis, whereas national studies include data from a diverse range of hospitals, each with varying neonatal care practices, resources, and healthcare infrastructures. Our tertiary care center, with its specialized neonatal unit, follows standardized protocols and benefits from experienced teams and access to advanced interventions, all of which likely contribute to improved survival rates. Additionally, selection bias may have influenced our findings, as our study included only neonates who received active resuscitation, whereas national datasets often include cases where palliative care was pursued instead of intensive management, particularly for neonates at the lowest gestational ages.

In lower-resource settings, mortality rates for extremely preterm neonates can vary widely but remain high. A retrospective follow-up of 505 preterm neonates admitted to the NICU at Jimma University Medical Center from 2017 to 2019 reported a 25.1% mortality rate, or 28.9 deaths per 1000 neonate-days, with comparable mortality rates observed in Nigeria (27.7%), Tigray (32.1%), Gondar (32.9 per 1000 neonate-days), and Addis Ababa (ranging from 25.3% to 36.4 per 1000 neonate-days) [4]. This study’s findings reaffirm the complexity of managing preterm neonates at the viability threshold, indicating that while advancements in neonatal care have led to improved outcomes in high-resource settings, mortality rates in lower-resource settings remain high due to disparities in the access to advanced medical interventions [18]. This aligns with the World Health Organization’s observation that survival rates for extremely preterm neonates can be markedly different depending on healthcare access, with high-income countries achieving over 90% survival rates for such neonates compared to a mere 10% in low-income settings [2]. Factors such as limited access to prenatal care, inadequate facilities for preterm birth management, limited numbers of healthcare professionals, and the scarcity of essential supplies like antenatal corticosteroids, warmth, infection control, and basic nutrition further exacerbate these disparities [7,19,20].

The reported mean duration of survival for deceased neonates, 30.5 days, along with a substantial standard deviation of 59.1 days reveals a striking variability in survival times within this vulnerable population. This wide range highlights the unpredictable and complex nature of outcomes for neonates born at the limits of viability. While the mean and standard deviation provide an overall picture of survival trends, the median survival time of 2 days and an interquartile range (IQR) of 0–23 days further underscore the disparities in outcomes. These findings indicate that half of the deceased neonates survived for just 2 days or less, with 25% passing away shortly after birth (0 days) and 25% surviving beyond 23 days. While some neonates succumbed almost immediately, others fought for significantly longer durations before passing, reflecting not only the inherent vulnerabilities of extreme prematurity but also the variability in their response to intensive medical interventions. Together, these statistics underline the profound challenges and uncertainties faced in neonatal intensive care and the critical need for individualized care strategies to improve outcomes.

Furthermore, our results, consistent with previous studies [3,5,21], demonstrate that the most critical morbidities that affect preterm neonates at the viability threshold are respiratory, infectious, and neurological complications; cardiovascular disorders; BPD; and ROP (Figure S1). These morbidities not only shape immediate outcomes but also influence long-term health, highlighting the need for focused interventions [22]. In particular, RDS was the most common morbidity affecting 71% to 94% of surviving neonates, with most requiring extensive respiratory support, including continuous positive airway pressure and conventional mechanical ventilation. The strong correlation between prolonged respiratory support and survival underscores the importance of effective lung management, despite the risks of chronic lung disease and BPD [23,24]. The effective management of RDS, including the use of surfactant therapy and mechanical ventilation, is crucial for improving survival rates [25]. In addition to respiratory support, caffeine therapy plays a vital role in managing apnea of prematurity. However, in our study, 21 neonates did not receive caffeine, as 16 of them died shortly after birth, preventing its administration. In the remaining neonates, the absence of a caffeine administration may be explained by a rapid clinical deterioration that precluded the timely initiation of pharmacological therapy. Among the neonates who did receive caffeine, a trend toward improved survival was observed, with a mortality rate of 51.9% compared to 66.7% among those who did not receive it. Nonetheless, this difference did not reach statistical significance (*p* = 0.250), suggesting that while caffeine administration may be associated with better outcomes, the effect could not be definitively confirmed in our sample. This finding is likely influenced by the high incidence of early deaths before treatment could be initiated. Therefore, although the benefits of caffeine in supporting respiratory function in preterm neonates are well documented, its impact on survival may have been underestimated in this study. Given the established benefits of caffeine therapy in preterm neonates—including reductions in BPD and severe brain injuries [26]—exploring strategies for even earlier administration, potentially initiated in the delivery room, may warrant further investigation [27]. These observations underscore the importance of early intervention and individualized care strategies that consider not only the gestational age and overall clinical status, but also the critical timing required for administering potentially beneficial therapies, such as caffeine.

Late-onset sepsis was the second most frequent complication (35.3%), highlighting the heightened infection risk due to immature immune systems. The third most prevalent morbidity was PDA, observed in 29.4% of the cases, emphasizing the challenges in managing hemodynamic stability in preterm neonates. Other morbidities occurred at lower rates, with BPD, ROP, IVH, PVL, and infections other than sepsis each affecting fewer than 25% of the neonates. These findings reinforce the complex health challenges faced by this population and the need for comprehensive, individualized management strategies.

Finally, our findings highlight the critical role of early and adequate nutrition in supporting the growth and immunity of preterm neonates, particularly through enteral feeding. The use of donor human milk and breastfeeding are linked to reduced rates of NEC and better neurodevelopmental outcomes [28,29]. Notably, the hospital’s milk bank serves as a crucial resource, providing optimal nutritional support, reducing infection risks, enhancing gastrointestinal health, and supporting long-term development in preterm neonates [30]. These findings align with current evidence advocating for the early initiation of enteral feeding in extremely preterm neonates to maximize health outcomes [31].

This study provides valuable insights into the morbidity and mortality of preterm neonates at the limits of viability in Greece, offering a regional perspective that contributes to both national and international neonatal care discussions. While limited by its retrospective, single-center design and relatively small sample size, it remains the first study of its kind in Greece, making it a useful reference for future research and healthcare policy development. The five-year data collection period strengthens the study by enabling the identification of clinical trends, while the logistic regression analysis highlights significant predictors of neonatal mortality, including gestational age, birth weight, and respiratory support, helping to contextualize current neonatal care practices and potential areas for improvement in Greece. However, the single-center scope may limit the generalizability, as outcomes could vary across different healthcare settings. It should also be noted that the authors encountered challenges in obtaining data from other hospitals, as these institutions were hesitant to grant approval for this study, possibly due to concerns about the dissemination of unfavorable data. This limitation confined the analysis to a single-center perspective, highlighting the need for improved collaboration to facilitate multicenter neonatal research in the future. Additionally, the retrospective design introduces potential biases related to data accuracy and completeness, and the lack of long-term follow-up data restricts the findings to short-term outcomes up to discharge. Finally, some potentially influential confounding variables, such as socioeconomic status, parental health, and environmental factors, were not included in the analysis but could impact neonatal outcomes. Given this study’s limitations, a national registry could be a valuable next step to assess long-term trends in morbidity and mortality across multiple centers in Greece, similarly to efforts in other countries [7], improving the generalizability of findings and guiding neonatal care strategies.

Improving outcomes for periviable neonates requires clear guidelines, effective communication, research, and data-driven analysis. Developing hospital-specific, multidisciplinary guidelines that integrate local resources and global best practices is essential, with regular updates to reflect emerging evidence. Antenatal counseling should be enhanced to provide empathetic, consistent, and detailed information, helping align parental expectations. Cross-institutional and longitudinal research can improve the understanding of long-term outcomes, while multicenter studies refine clinical practices across diverse settings. Establishing robust tracking systems for survival rates, morbidities, and developmental progress will help optimize care protocols. By prioritizing research, collaboration, and innovation, hospitals can advance neonatal care and improve outcomes for this vulnerable population.

## 5. Conclusions

This study underscores the urgent need for targeted health policy interventions to improve outcomes for preterm neonates born at the limits of viability. While the gestational age and birth weight remain primary determinants of survival, our findings also highlight the impact of modifiable clinical factors—such as antenatal corticosteroid use, surfactant therapy, early respiratory support, and enteral nutrition—on neonatal mortality and morbidity. These results support the importance of implementing evidence-based protocols that ensure the timely and consistent delivery of critical interventions across perinatal care settings.

As the first study of its kind in Greece, these findings provide a foundation for informing national neonatal health strategies. The development of standardized clinical pathways, investment in neonatal infrastructure and specialized workforce training, and the improved coordination of interhospital transfers are essential steps toward enhancing care for extremely preterm neonates. Furthermore, the establishment of a national neonatal registry would enable systematic data collection and quality monitoring, supporting evidence-informed policies and long-term improvements in neonatal outcomes across the country.

## Figures and Tables

**Table 1:** Maternal and neonatal characteristics.

		N	%
Mother’s age, Mean (SD)		31.1 (7.1)	
Birth order	1	40	54.8
	2	20	27.4
	3	10	13.7
	4	3	4
Gestational age, Mean (SD), Median (IQR)		24.1 (1.3)	24.3 (22.9–25.2)
Number of fetuses	1	60	82.2
	2	10	13.7
	3	3	4.1
Sex	Male	39	53.4
	Female	34	46.6
IVF	No	67	91.8
	Yes	6	8.2
Type of delivery	Vaginal	38	52.1
	Cesarean Section	35	47.9
Prenatal corticosteroids	Complete treatment	27	37.0
	Partial treatment	18	24.7
	None	28	38.4
Apgar score 1, Median (IQR)		4.0 (2–5)	
Apgar score 5, Median (IQR)		7.0 (4–8)	
Birth weight, Mean (SD)		603.6 (155.1)	
Head circumference at birth, Mean (SD)		22.1 (1.7)	
Management in the operating or delivery room			
NEOPUFF		45	63.4
Intubation		44	62.0
Adrenaline		12	16.9
Other		1	1.4

IQR = interquartile range; IVF = in vitro fertilization; and SD = standard deviation.

**Table 2:** Neonatal clinical characteristics during hospitalization.

			N	%
Late sepsis		No	65	89.0
		Yes	8	11.0
Possible sepsis (occurrences)		1	3	42.9
		2	1	14.3
		3	2	28.6
		4	1	14.3
Other infection		No	63	86.3
		Yes	10	13.7
NEC		Intestinal Perforation	1	1.4
PDA		Paracetamol	10	71.4
		Paracetamol, Ibuprofen	1	7.1
		Ibuprofen	3	21.4
Cerebral hemorrhage (Papille)— left side (Grade)		1	2	40.0
		2	1	20.0
		3	1	20.0
		4	1	20.0
Cerebral hemorrhage (Papille)—right side (Grade)		1	1	20.0
		2	3	60.0
		4	1	20.0
PVL—left side (Grade)		1	1	100.0
ROP—left side eye (Stage)		1	1	25.0
		2	2	50.0
		4	1	25.0
ROP—right side eye (Stage)		1	1	20.0
		2	3	60.0
		4	1	20.0
Laser photocoagulation—left		Yes	1	1.4
		No	72	98.6
Laser photocoagulation—right		Yes	1	1.4
		No	72	98.6
			Median (IQR)	
Length of hospital stay (days)			6 (1–58)	
Duration of survival of the deceased neonates (days)			2 (0–23)	
			Mean (SD)	
Respiratory support (days)	NCPAP		5 (6.2)	
	Conventional (CMV, PTV, and SIMV)		20.2 (19.4)	
	HFO		13.7 (15.7)	
	HOOD		3 (5.2)	
	Continuous Oxygen		4 (5.3)	
Duration of survival of the deceased neonates (days)			30.5 (59.1)	

CMV = continuous mandatory ventilation; GA = gestational age; HFO = high flow oxygen; IQR = interquartile range; NCPAP = nasal continuous positive airway pressure; NEC = necrotizing enterocolitis; PDA = patent ductus arteriosus; PTV = patient-triggered ventilation; PVL = periventricular leukomalacia; ROP = retinopathy of prematurity; SD = standard deviation; and SIMV = synchronized intermittent mandatory ventilation.

**Table 3:** Results from univariate analysis for death.

	Death					
	No			Yes		
	Mean (SD)		Median (IQR)	Mean (SD)	Median (IQR)	p
Maternal age	32.3 (7.1)		-	30.1 (7.1)	-	0.193++
Gestational age	24.9 (1)		25.1 (24.5–25.6)	23.4 (1.2)	23.3 (22.4–24.3)	<0.001+
Apgar score at 1st minute	4.4 (1.9)		5 (3–6)	3.3 (2.3)	3 (1–5)	0.099+
Apgar score at 5th minute	6.6 (1.9)		7 (5–8)	5.2 (2.5)	5 (3–8)	0.047+
Birth weight	684.4 (134.7)		-	537.3 (139.6)	-	<0.001++
BE	7.9 (5.5)		-	11.1 (8.9)	-	0.085+
Respiratory support—NCPAP (days)	5 (6.2)		1.5 (0–9)	0.7 (2.8)	0 (0–0)	<0.001+
Respiratory support—Conventional (CMV, PTV, and SIMV) (days)	20.2 (19.4)		18.5 (1–30)	3.5 (4.3)	2 (0–5)	<0.001+
Respiratory support—HFO (days)	13.7 (15.7)		8 (0–20)	1.7 (5.3)	0 (0–0)	<0.001+
Respiratory support—HOOD (days)	3 (5.2)		0 (0–4)	0.2 (0.9)	0 (0–0)	<0.001+
Plasma transfusions (times)	0.3 (0.7)		0 (0–0)	0.5 (1.1)	0 (0–0)	0.348+
Red cell complement transfusions (times)	3.7 (3.9)		3 (0–6)	0.7 (1.1)	0 (0–1)	0.002+
Length of hospital stay (days)	57.6 (48.4)		69.5 (1–91)	5.7 (9.7)	2 (1–7)	<0.001+
		Ν	%	Ν	%	
Birth order	1	16	40.0	24	60.0	0.750÷
	2	10	50.0	10	50.0	
	≥3	6	46.2	7	53.8	
Number of fetuses	1	23	38.3	37	61.7	0.042÷
	>1	9	69.2	4	30.8	
Sex	Male	16	41.0	23	59.0	0.604÷
	Female	16	47.1	18	52.9	
Type of delivery	Vaginal	15	39.5	23	60.5	0.434÷
	Cesarean section	17	48.6	18	51.4	
Prenatal corticosteroids	Complete treatment	10	37.0	17	63.0	0.467÷
	Partial treatment	10	55.6	8	44.4	
	None	12	42.9	16	57.1	
NEOPUFF	No	9	34.6	17	65.4	0.178÷
	Yes	23	51.1	22	48.9	
Intubation	No	13	48.1	14	51.9	0.683÷
	Yes	19	43.2	25	56.8	
Adrenaline	No	24	40.7	35	59.3	0.099÷
	Yes	8	66.7	4	33.3	
Surfactant administration	No	9	31.0	20	69.0	0.021÷
	1 dose	8	36.4	14	63.6	
	2 or 3 doses	15	68.2	7	31.8	
Enteral nutrition	No	15	29.4	36	70.6	<0.001÷
	Yes	17	77.3	5	22.7	
Other infection	No	26	41.3	37	58.7	0.317÷÷
	Yes	6	60.0	4	40.0	
Caffeine administration	No	7	33.3	14	66.7	0.250÷
	Yes	25	48.1	27	51.9	
PDA	No	24	40.7	35	59.3	0.264÷
	Yes	8	57.1	6	42.9	
Red cell transfusion in the first three days	No	17	41.5	24	58.5	0.644÷
	Yes	15	46.9	17	53.1	

+ Mann–Whitney test; ++ Student’s *t*-test; ÷ Pearson’s exact test, ÷÷ Fisher’s exact test; PDA = patent ductus arteriosus; and BE = base excess.

**Table 4:** Logistic regression analysis of maternal and neonatal factors associated with neonatal mortality.

	OR (95% CI) +	*p*
Birth weight	0.99 (0.98–0.99)	0.029
Gestational age	0.38 (0.20–0.71)	0.003

+ OR: odds ratio.

**Table 5:** Logistic regression analysis of neonatal-specific factors associated with neonatal mortality.

	OR (95% CI) +	*p*
Gestational Age	0.25 (0.12–0.52)	<0.001
Respiratory Support—Conventional (CMV, PTV, SIMV) (days)	0.87 (0.79–0.96)	0.005

+ OR: odds ratio.

## Data Availability

The original contributions presented in this study are included in the article/Supplementary Material. Further inquiries can be directed to the corresponding author.

## Supplementary Materials

The following supporting information can be downloaded at https://www.mdpi.com/article/10.3390/life15050708/s1: Figure S1: Morbidities among surviving neonates.

## Abbreviations

The following abbreviations are used in this manuscript:

BE	Base excess
BPD	Bronchopulmonary dysplasia
CI	Confidence interval
CMV	Continuous mandatory ventilation
GA	Gestational age
HFO	High flow oxygen
IQR	Interquartile range
IVF	In vitro fertilization
IVH	Intraventricular hemorrhage
NCPAP	Nasal continuous positive airway pressure
NEC	Necrotizing enterocolitis
NICU	Neonatal Intensive Care Unit
OR	Odds ratio
PDA	Patent ductus arteriosus
PTV	Patient triggered ventilation
PVL	Periventricular leukomalacia
RDS	Respiratory distress syndrome
ROP	Retinopathy of prematurity
SD	Standard deviation
SIMV	Synchronized intermittent mandatory ventilation
