# Supplementary figures and images for: Morbidity and Mortality Trends in Preterm Neonates at the Limits of Viability: Retrospective Observations from One Greek Hospital
Source: Life (Basel). 2025 Apr 27;15(5):708. doi: 10.3390/life15050708 (PMC12112811; doi:10.3390/life15050708)

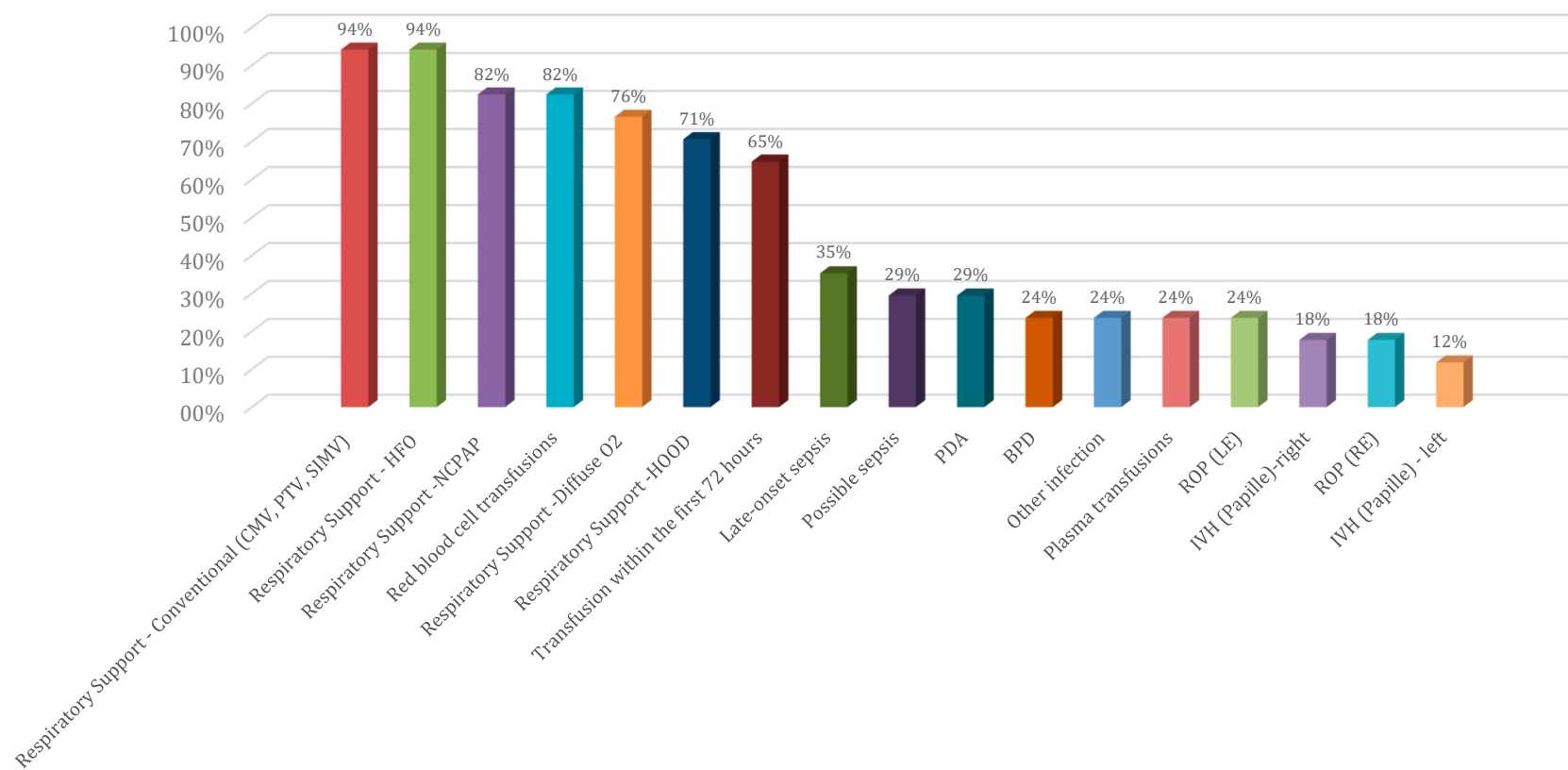

Figure S1. Morbidities among surviving neonates

Supplement: Supplementary file 1 [file life-15-00708-s001.zip › life-3511521-supplementary.pdf]
